# Preparations of Tough and Conductive PAMPS/PAA Double Network Hydrogels Containing Cellulose Nanofibers and Polypyrroles

**DOI:** 10.3390/polym12122835

**Published:** 2020-11-28

**Authors:** Cheng-Wei Tu, Fang-Chang Tsai, Jem-Kun Chen, Huei-Ping Wang, Rong-Ho Lee, Jiawei Zhang, Tao Chen, Chung-Chi Wang, Chih-Feng Huang

**Affiliations:** 1 Industrial Technology Research Institute, Chutung, Hsinchu 31057, Taiwan; CWTu@itri.org.tw; 2 Hubei Key Laboratory of Polymer Materials, Key Laboratory for the Green Preparation and Application of Functional Materials (Ministry of Education), Hubei Collaborative Innovation Center for Advanced Organic Chemical Materials, School-Soaked of Materials Science and Engineering, Hubei University, Wuhan 430062, China; 3 Department of Materials Science and Engineering, National Taiwan University of Science and Technology, Taipei 10607, Taiwan; jkchen@mail.ntust.edu.tw; 4 Department of Chemical Engineering, i-Center for Advanced Science and Technology (iCAST), National Chung Hsing University, Taichung 40227, Taiwan; ac9562@gmail.com (H.-P.W.); rhl@nchu.edu.tw (R.-H.L.); 5 Key Laboratory of Marine Materials and Related Technologies, Zhejiang Key Laboratory of Marine Materials and Protective Technologies, Ningbo Institute of Materials Technology and Engineering, Chinese Academy of Sciences, Ningbo 315201, China; zhangjiawei@nimte.ac.cn (J.Z.); tao.chen@nimte.ac.cn (T.C.); 6 Division of Cardiovascular Surgery, Veterans General Hospital, Taichung 40705, Taiwan; chungchi@vghtc.gov.tw

**Keywords:** double network hydrogels, conductive hydrogels, PAMPS, PAA, TEMPO-oxidized cellulose nanofibers, polypyrroles

## Abstract

To afford an intact double network (sample abbr.: DN) hydrogel, two-step crosslinking reactions of poly(2-acrylamido-2-methylpropanesulfonic acid) (i.e., PAMPS first network) and then poly(acrylic acid) (i.e., PAA second network) were conducted both in the presence of crosslinker (*N*,*N*′-methylenebisacrylamide (MBAA)). Similar to the two-step processes, different contents of 2,2,6,6-tetramethyl-1-piperidinyloxy (TEMPO) oxidized cellulose nanofibers (TOCN: 1, 2, and 3 wt.%) were initially dispersed in the first network solutions and then crosslinked. The TOCN-containing PAMPS first networks subsequently soaked in AA and crosslinker and conducted the second network crosslinking reactions (TOCN was then abbreviated as T for DN samples). As the third step, various (T–)DN hydrogels were then treated with different concentrations of FeCl_3(aq)_ solutions (5, 50, 100, and 200 mM). Through incorporations of ferric ions into (T–)DN hydrogels, notably, three purposes are targeted: (i) strengthen the (T–)DN hydrogels through ionic bonding, (ii) significantly render ionic conductivity of hydrogels, and (iii) serve as a catalyst for the forth step to proceed with in situ chemical oxidative polymerizations of pyrroles to afford polypyrrole-containing (sample abbr.: Py) hydrogels [i.e., (T–)Py–DN samples]. The characteristic functional groups of PAMPS, PAA, and Py were confirmed by FT–IR. Uniform microstructures were observed by cryo scanning electron microscopy (cryo-SEM). These results indicated that homogeneous composites of T–Py–DN hydrogels were obtained through the four-step process. All dry samples showed similar thermal degradation behaviors from the thermogravimetric analysis (TGA). The T_2_–Py_5_–DN sample (i.e., containing 2 wt.% TOCN with 5 mM FeCl_3(aq)_ treatment) showed the best tensile strength and strain at breaking properties (i.e., σ_Tb_ = 450 kPa and ε_Tb_ = 106%). With the same compositions, a high conductivity of 3.34 × 10^−3^ S/cm was acquired. The tough T_2_–Py_5_–DN hydrogel displayed good conductive reversibility during several “stretching-and-releasing” cycles of 50–100–0%, demonstrating a promising candidate for bioelectronic or biomaterial applications.

## 1. Introduction

Materials with soft and ductile properties are ideal for flexible biomaterials or devices due to their great deformability under stress. As one of the well-known soft materials, polymeric hydrogels could absorb and retain large amounts of water in their three-dimensional networks. Polymeric hydrogels have thus achieved a considerable amount of applications, such as drug delivery systems [1,2], super-absorbents [3,4,5], microfluidics [6,7], sensors [8,9,10], and actuators [11,12,13,14]. However, conventional hydrogels generally represent poor mechanical strength and brittle properties after fully swelling, which restricts their extensive uses. In the last two decades, many efforts have been devoted to enhancing the strength and toughness of hydrogels. To facilitate the applications requiring high mechanical properties, double network (DN) hydrogels [15], nanocomposite hydrogels [16,17], and microgel-reinforced hydrogels [18,19] were presented. Among them, Gong and coworkers reported an unprecedented tough DN hydrogels system consisting of stiff/brittle and ductile/soft chain in 1st and 2nd interpenetrating network structures, respectively [15]. The extraordinarily mechanical properties of DN hydrogels were mainly due to the uniform penetrated networks which contributed the effective energy dissipation from the 1st stiff/brittle network to the 2nd ductile/soft network. Afterward, a large amount of strong DN hydrogels was developed and successfully expanded the potential applications of hydrogels [20,21].

Recently, hydrogels incorporated with inherently conductive polymers such as poly(3,4-ethylenedioxythiophene) (PEDOT) [22,23,24], polyaniline (PANI) [25,26,27], and polypyrrole (Py) [28,29] aroused much attention due to their potential in the field of flexible electronics [30] and flexible energy storage systems [31,32]. One main advantage of conductive polymers compared to other conductors is the tunable intrinsic electrical properties controlled by the organic synthesis procedure, such as molecular weights, functional groups, and dopants. Among the numerous conductive polymers, Py is by far the most extensively studied because of its good conductivity, easy synthesis, good environmental stability, and low toxicology [28,29,33]. The rigid structure of the Py backbone is the main drawback, which leads to poor solubility and subsequently limits its practical uses. In this circumstance, a simple oxidative method to carry out in situ polymerization with water-soluble monomers and oxidizers provides a feasible way to obtain conductive hydrogels. However, a low-conductivity feature remains a problematic challenge.

On the other hand, celluloses extracted from plant-based biomass or synthesized by non-plant sources are one of the most naturally ubiquitous and abundant polysaccharides. Two D-glucose rings connected via a β–1,4 glycosidic linkage [34] are generally known to provide the repeating saccharide unit of cellulose. Due to strong intermolecular hydrogen bonding between the backbones of the main chain, the polysaccharide chains aggregate to form micro-fibrils with high crystallinity. The well-packed hierarchical microscale fibers thus possess excellent tensile strength (ca. 300 MPa) and Young’s modulus (ca. 10 GPa). However, the large bundles of celluloses make it difficult for the solution or melting compounding process to attain uniform polymeric composites. Isogai et al. [35] used 2,2,6,6–tetramethylpiperidine–1–oxy (TEMPO) compound to effectively oxidize the neutral surfaces of microscale cellulose fibers to negatively charged surfaces (i.e., carboxylate groups). Numerous negative charges on the surfaces provide significant repulsion forces for disintegration and obtain cellulose nanofibers. The diameters of the so-called TEMPO-oxidized cellulose nanofiber (TOCN) typically are under 10 nm and high aspect ratio. Additionally, the repulsions among surface carboxylate groups promote TOCNs to disperse in aqueous or high-polar organic solvents. In recent studies, TOCNs were hybridized with the matrix to construct a porous 3D framework for fabricating TOCN/hydrogels [36,37,38,39] or TOCN/polymer composites [40,41,42,43,44]. For example, Sultana et al. fabricated TOCN-containing thermo-responsive hydrogels to efficiently inhibit post-surgical peritoneal adhesions [37]. Li et al. incorporated TOCN into polyacrylamide-based shape memory hydrogels to improve the mechanical properties [45]. Recently, TOCN was further incorporated into conductive hydrogel systems and revealed a good enhancement for their electrical properties. Lin et al. reported conductive sodium alginate (SA) hydrogels with TOCN, fabricated through the formation of crosslinks with calcium ions and in situ polymerization of pyrrole. The conductivity of the SA gel was successfully enhanced with the increase of TOCN content. About ten-times-higher conductivity was acquired at 5 wt.% of TOCN content (8.9 × 10^−3^ S/cm) compared to the SA gel containing no TOCN (8.2 × 10^−4^ S/cm) [33]. The mechanical properties were also enhanced with increasing TOCN content and reached to 1.02 MPa of compression stress and 75% of fracture strain. Liu et al. reported oxalic acid crosslinked conductive polyaniline (PANI)/TOCN hydrogel has a high areal specific capacitance [46]. However, to the best of our knowledge, studies about hydrogels with good conductivity and mechanical properties are still rare.

**Scheme 1:**
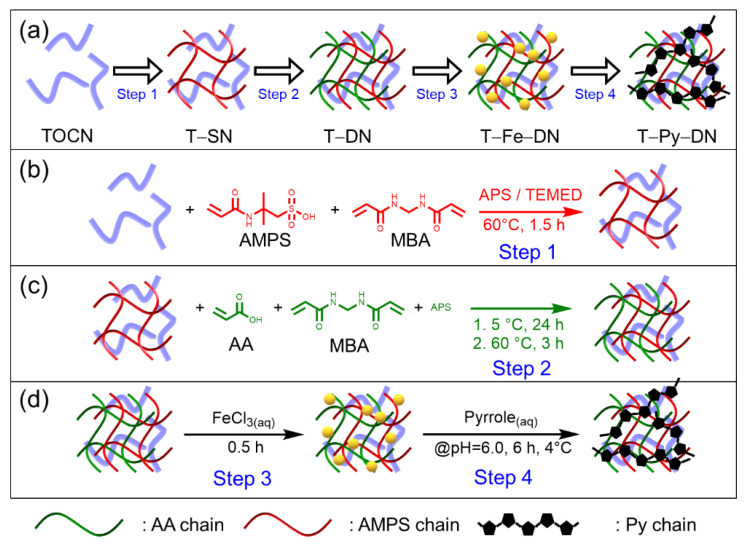
(**a**) Four steps of stratagem for the preparations of T–Py–DN hydrogels. (**b**) Poly(2-acrylamido-2-methylpropanesulfonic acid (PAMPS) single network (SN) hydrogels hybridized with 2,2,6,6-tetramethyl-1-piperidinyloxy oxidized cellulose nanofibers (TOCN) (i.e., T–SN). (**c**) PAMPS/ poly(acrylic acid)(PAA) DN hydrogels hybridized with TOCN (i.e., T–DN). (**d**) conductive PAMPS/PAA DN hydrogels hybridized with TOCN and polypyrrole (Py)-controlling hydrogels (i.e., T–Py–DN).

In this work, a series of tough and conductive DN hydrogels hybridized with TOCN (note: TOCN was then abbreviated as T for DN samples) and Py (i.e., T–Py–DN hydrogels) were fabricated through a four-step process (Scheme 1): (1) crosslinking reactions of homogeneous first network solutions, including 2-acrylamido-2-methylpropanesulfonic acid (AMPS), *N,N’*-methylenebisacrylamide (MBAA), and ammonium persulfate/tetramethylethylenediamine (APS/TMEDA), were conducted with or without TOCNs to obtain a single network of (T–)SN hydrogels. (2) Through redox-induced radical polymerizations, again, the (T–)SNs were immersed in 2nd network solutions, including acrylic acid (AA) monomers, MBAA, and APS to afford (T–)DN hydrogels. (3) Various (T–)DN hydrogels were then treated with different concentrations of FeCl_3(aq)_ solutions to incorporate ferric ions into (T–)DN hydrogels. (4) The Fe-treated (T–)DN hydrogels were soaked in pyrroles and further carried out in situ chemical oxidative polymerizations to attain polypyrrole-containing (sample abbr.: Py) conductive hydrogels [i.e., (T–)Py–DN]. The resulting (T–)Py–DN hydrogels were characterized by FT–IR and UV–Vis spectroscopy. The microstructures, thermal, mechanical, and electrical properties of various hydrogels were examined by cryo scanning electron microscopy (cryo-SEM), thermogravimetric analysis (TGA), universal tensile machine, and four-point probe sheet resistivity system, respectively. Eventually, the electrical resistance of T–Py–DN hydrogel over several elongation cycles were investigated to confirm the stability of the hydrogel while applying an external force.

## 2. Materials and Methods

### 2.1. Materials

Paper pulp used in this study was kindly supported by Chung-Hua Paper Inc. (Hualien, Taiwan). Hypochlorite (NaClO, 12%), sodium hydroxide (NaOH, 96%), sodium bromide (NaBr, >99%), sodium ammonium persulfate (APS, 98%), hydrochloric acid (HCl, 35%), and potassium bromide (KBr, 99%) were purchased from Showa Corporation (Saitama, Japan). Acrylic acid (AA, 98%) and iron (III) chloride hexahydrate (FeCl_3_·6H_2_O, 99%), were purchased from ACROS Organics (Geel, Belgium). 2,2,6,6-tetramethylpiperidine 1-oxyl (TEMPO, 98%+), 2-acrylamido-2-methylpropane sulfonic acid (AMPS, 98%), *N*,*N*′-methylenebisacrylamide (MBAA, 97%), pyrrole (Py, 98%), *N,N,N’,N’*-tetramethylethylenediamine (TMEDA, 99%), and ethylenediaminetetraacetic acid (EDTA, 99%) were used as received from Alfa Aesar (MA, US) without further purification.

### 2.2. Preparations of TEMPO-Oxidized Cellulose Nanofibers (TOCNs)

First, the impurities in paper pulps were removed by dispersing them in DI water for several hours and the suspended pulps were collected after filtrating. According to previous study [40,42,47], second, freshly cleaned pulps (10.0 g), NaBr (0.16 g), and TEMPO (1.0 g) were dispersed in 1 L DI water with vigorous stirring. The reaction was gradually turning to light orange color after TEMPO was fully dissolved and then NaClO solution (62.3 g) was then fed to the mixture. The pH value of the semi-batch reactor was controlled at ca. 10 by continuously feeding 1.0 M NaOH_(aq)_. With a desired period, the reaction suspensions were washed to decrease the pH to ca. 7.5. The crudes were collected through centrifugation and dried out by freeze-fried method to avoid the aggregation (yield 87.6%).

### 2.3. Preparation of Single Network Hydrogels Included with TOCN (i.e., T–SN Hydrogel)

AMPS and MBAA were used as monomer and crosslinker for the 1st network, respectively. AMPS (1.46 g, 7.1 mmol), MBAA (0.046 g, 0.3 mmol), and a desired amount of TOCN were vigorously mixed in water (6 mL). With additions of 1 wt.% solution of TMEDA (0.22 mL, 0.015 mmol) and 10 wt.% solution of APS (0.17 mL, 0.11 mmol), the reaction mixture was injected into a reaction holder made by two glass plates with a space of 1.5 mm. Then, the 1st network hydrogel was prepared by placing the reaction holder at 60 °C for 1.5 h. The unreacted compounds were removed by dipping the prepared T–SN in a large amount of fresh DI water for 1 day.

### 2.4. Preparation of Double Network Hydrogels Included with TOCN (i.e., T–DN Hydrogels)

The obtaining T–SN hydrogels were immersed in a mixture with AA (9.96 mL, 101.5 mmol), MBAA (0.016 g, 0.1 mmol), APS (0.3 g, 1.3 mmol), and water (28 mL). The mixture was kept in a refrigerator for 1 day to reach an equilibrium absorption of 2nd network reactants. DN hydrogel was then obtained through keeping the swollen T–SN hydrogel in a reaction holder made by two glass plates with a space of 1.5 mm at 60 °C for 3 h. The unreacted compounds were also removed by immersing the DN hydrogel in a large amount of fresh DI water for 1 day.

### 2.5. Preparation of Double Network Hydrogels Hybridized with TOCN and Py (i.e., from T–Fe–DN to T–Py–DN Hydrogels)

The fresh-cleaned T–DN hydrogels were dipped in FeCl_3(aq)_ solutions with concentrations of 5, 50, 100, or 200 mM for 0.5 h unless otherwise stated. Subsequently, the Fe-treated T–DN hydrogels (i.e., T–Fe–DN samples) were transferred into a 0.3 M pyrrole aqueous. After turning the reaction solution to pH = 6 by 0.1 N HCl_(aq)_, the oxidative polymerization process was kept in the refrigerator at 4 °C for another 6 h. Again, the unreacted monomers and impurities were removed by immersing the DN hydrogels in a large amount of fresh DI water for 1 day.

### 2.6. Determination of the Carboxylate Content (CC) and Degree of Oxidation (DO) of TOCN

Conductometric titration (CT) is used to determine the carboxylate content (CC) and degree of oxidation (DO) for the freshly prepared TOCN. The pH value of a diluted TOCN aqueous suspension was first acidified by HCl_(aq)_ from neutral to pH = ca. 2.5 and then titrated by aqueous solution of 0.05 M NaOH_(aq)_. The DO of TOCN can be calculated with the following equation: DO = 162 × n × COOH/(W–14 × n × COOH). W is the total weight of TOCN in the aqueous suspension. “n × COOH” is the mole number of carboxylic acid in TOCNs, which is estimated from the titrated amounts of NaOH_(aq)_.

### 2.7. Estimations of Water Content and Degree of Swelling for Hydrogels

Hydrogels were immersed to a large amount of water and allowed to free swelling for 1 day. After measuring the weight of swollen hydrogels, the samples were then dried in an oven at 105 °C for 1 day. The water content and degree of swelling were calculated by the following equations:

$$\mathrm{water}\;\mathrm{content}\;(\%)\;=\;\frac{w-w_{dry}}{w}\times 100\%$$

$$\mathrm{degree}\;\mathrm{of}\;\mathrm{swelling}\;=\;\frac{w-w_{dry}}{w_{dry}}$$

where *w* denotes the weight of hydrogels after fully swelling and *w_dry_* denotes the dry weight of hydrogels.

### 2.8. Establishment of the Calibration Curve of Fe(III)EDTA Solution and Estimations of Fe^3+^ Concentration Absorbed in Hydrogels

An amount of 0.125 mM Fe(III)EDTA solution was prepared via mixing equal amounts of 250 mM FeCl_3(aq)_ and 250 mM EDTA_(aq)_ solutions. Then, dilutions of Fe(III)EDTA aqueous solution (0.0625, 0.0313, 0.0156, and 0.0078 mM) were prepared from 0.125 mM Fe(III)EDTA solution with a desired amount of DI water. The calibration curve was obtained by fitting the plot of UV–Vis absorbance (at 260 nm) versus concentration of Fe(III)EDTA aqueous solution as shown in Figure S1 (in supplementary materials). To determine the Fe^3+^ concentration in hydrogels, the residual solution on the surface of FeCl_3(aq)_ treated sample was removed by tissue paper and the hydrogel samples with volume around 0.15 cm^3^ was subsequently immersed in 50 mL of 0.2 M EDTA_(aq)_ solution for 120 h. By comparing the absorbance intensity at 260 nm to the calibration curve, the concentration of extracted Fe(III)EDTA aqueous solution was obtained. Thus, the Fe^3+^ concentration in hydrogels could be calculated through the total molar number of Fe^3+^ calculated from the extracted Fe(III)EDTA aqueous solution dividing by the volume of the hydrogels.

### 2.9. Characterization

Fourier-transform infrared (FT–IR) spectra of TOCN and dried hydrogels were recorded by an attenuated total refraction FT–IR Spectrometer (PerkinElmer FT–IR SP-1, Waltham, MA, USA). The surface morphologies of the paper pulp, TOCN, or hydrogels were observed by thermal field emission scanning electron microscope with a cryo system (FE-SEM JEOL JSM-7800F/Quorum Technologies PP3010T Cryo-SEM Preparation System, Lewes, UK). A universal testing machine (Cometech QC-513A2) was used for the tensile test of the dumbbell-shaped hydrogel with the gauge length, width, and thickness are 50 mm, 4 mm, and 2.5 mm, respectively. The tensile speed was controlled at 25 mm/min during the test. Thermal gravimetric analyses (TGA) were performed under N_2_ using a Thermo Scientific Cahn TGA Versa Therm-HS at a heating rate of 10 °C/min and recorded the weight data from 120 to 800 °C. The thermal decomposition temperature (*T*_d,5%_) was taken as the 5% weight loss of the dried sample during heating. The UV–Vis absorption spectra of hydrogels was measured by a UV–Visible spectrophotometer (Thermo Scientific™ BioMate™ 3 Series Spectrophotometers, Waltham, MA, USA). The electrical conductivity of the hydrogel was measured using the four-point probe method with the model of LORESTA-GP MCP-T600/ESP type. The electrical resistance of T–Py–DN hydrogels during “stretching-and-releasing” tests was measure by GWINSTEK LCR-6100 LCR meter.

## 3. Results and Discussion

### 3.1. Preparations of TOCN and Hybridizations with Double Network (i.e., T–DN) Hydrogels

**Figure 1:**
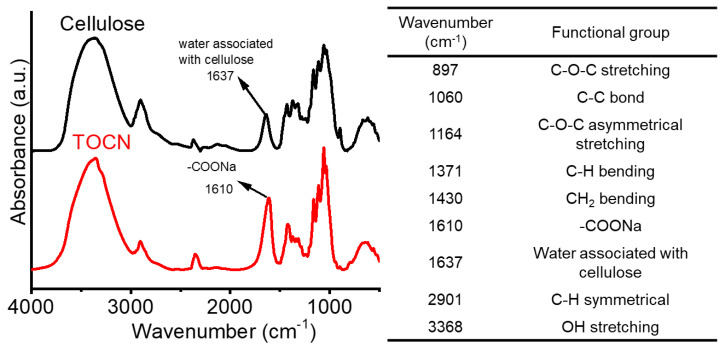
FT–IR spectra of original cellulose and TOCN and characteristic signals.

The oxidation agent of 2,2,6,6-tetramethylpiperidine 1-oxyl (TEMPO) was used to dismantle paper pulps to afford TEMPO-oxidized cellulose nanofibers (TOCNs) according to the previous studies [47]. Notably, selective oxidation of the C6-hydroxyl group on glucose units occurs during the reaction and produces carboxylic acid sodium salt groups. Through the conductivity titration method, the degree of oxidation (DO) and carboxylic acid content (CC) of our obtaining TOCN were thus estimated at 0.46 per 3 hydroxyl groups on the glucose repeating units and 2.03 mmole/g, respectively. The carboxylic acid sodium salt groups on TOCN can be characterized by FT–IR analysis. As shown in Figure 1, we can acquire the characteristic peak of the original cellulose in paper pulps associated with bonded water (i.e., 1637 cm^−1^, medium). In the case of TOCN, the characteristic peak of carboxylate group was observed (i.e., 1610 cm^−1^, strong). As displayed in Figure S2 (in supplementary materials), the mean diameter of cellulose fibers was reduced from 12.8 ± 4.1 µm to 15 ± 3 nm after the TEMPO oxidation reaction. We thus successfully prepared TOCNs with high values of DO and CC.

**Figure 2:**
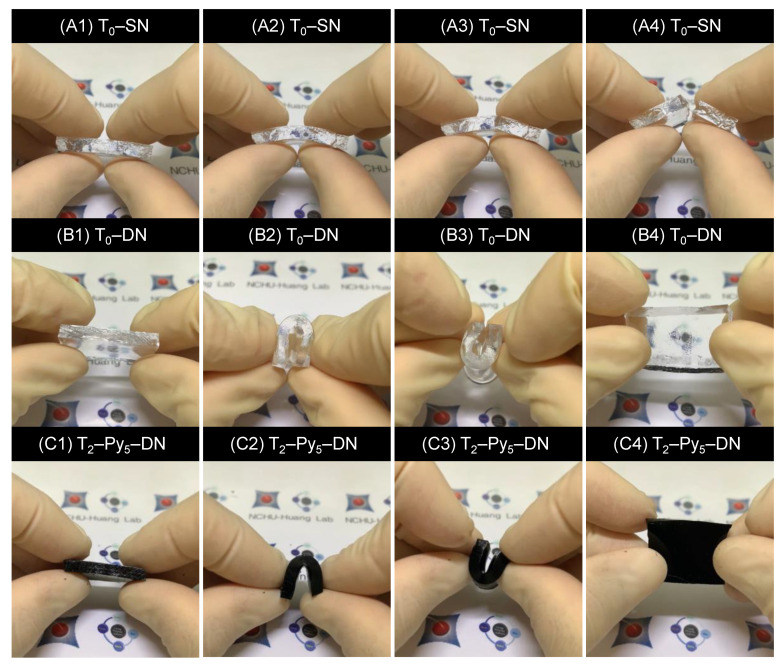
Appearances and cyclic hand-bending tests of (**A**) T_0_–SN, (**B**) T_0_–DN, and (**C**) T_2_–Py_5_–DN hydrogels.

To obtain conductive hydrogels, Scheme 1a depicts our overall strategy for the preparations of hydrogels hybridized with various TOCN contents, including single network (named as T–SN), double network (i.e., T–DN), FeCl_3(aq)_ treated T–DN (i.e., T–Fe–DN), and final polypyrrole-containing double network (i.e., T–Py–DN) hydrogels. The composite hydrogels were prepared by a four-step process: In Step 1 shown in Scheme 1b, crosslinking reactions of homogeneous first network solutions, comprising 2-acrylamido-2-methylpropanesulfonic acid (AMPS), *N*,*N*′-methylenebisacrylamide (MBAA), and ammonium persulfate/tetramethylethylenediamine (APS/TMEDA), were conducted without or with TOCNs (1, 2, and 3 wt.%) to obtain a single network of (T–)SN hydrogels. In Step 2 shown in Scheme 1c, the (T–)SNs were immersed in 2nd network solutions, comprising acrylic acid (AA), MBAA, and APS, to afford (T–)DN hydrogels through the same redox-induced radical polymerization mechanism. In Step 3 shown in Scheme 1d, various (T–)DN hydrogels were then treated with different concentrations of FeCl_3(aq)_ solutions (5, 50, 100, and 200 mM) to incorporate ferric ions into (T–)DN hydrogels. In Step 4 shown in Scheme 1d, the Fe-treated samples [i.e., (T–)Fe–DN] were soaked in pyrroles and further carried out in situ chemical oxidative polymerizations to attain polypyrroles-containing (sample abbr.: Py) conductive hydrogels [i.e., (T–)Py–DN]. The hydrogel samples were abbreviated by T_a_–DN, T_a_–Fe_b_–DN, and T_a_–Py_b_–DN, where a denotes the TOCN feeding weight percent comparing to the AMPS monomer and b represents the immersing concentrations of FeCl_3(aq)_ for T_a_–DN hydrogels. Detailed information was summarized in Table 1. The appearances and cyclic hand-bending tests of T_0_–SN, T_0_–DN, and T_2_–Py_5_–DN hydrogels were shown in Figure 2. T_0_–SN hydrogel broke easily while bending, but DN and T–Py–DN hydrogels revealed good bendability. These results indicated significant improvement of the mechanical property of DN-type hydrogels. Meanwhile, the introduction of Py chains only revealed color changes to black but did not significantly affect the mechanical property.

### 3.2. Incorporations of Various Fe^3+^ Concentrations into T–DN Hydrogels

To compose conductive DN hydrogels, different (T–)DNs were then immersed in different concentrations of FeCl_3(aq)_ solutions. This incorporating step encloses three purposes: (i) strengthen the (T–)DN hydrogels through ionic bonding of carboxylates and ferric ions, (ii) significantly render ionic conductivity of hydrogels with the aids of ferric ions, and (iii) catalyze the next step of in situ chemical oxidative polymerizations of pyrroles by ferric ions. Eventually, polypyrroles-containing conductive hydrogels can be attained. An effective chelating agent of ethylenediaminetetraacetic acid (EDTA) was adopted to extracting Fe^3+^ within T–Fe–DNs through the formation of stable Fe(III)EDTA complex. After extractions of T–Fe–DNs, the Fe^3+^ concentrations in the resulting solutions can be estimated by UV–Vis spectroscopy. We acquired a calibration line from various Fe(III)EDTA concentrations at the wavelength of 260 nm (in supplementary materials, Figure S1b). As shown in Figure S3 (in supplementary materials), accordingly, we can estimate the concentrations of ferric ions and summarize in Table 2. Rationally, the higher immersing [FeCl_3_]_0_ or longer immersing time led to the higher Fe^3+^ contents within the T–Fe–DN hydrogels. The apparent color changes of various T–Fe–DN hydrogels treated with FeCl_3_ solutions and after removing Fe^3+^ by ETDA solutions were shown in Figure S4 (in supplementary materials). As displayed in Figure S4a, the color changes of T–Fe–DN hydrogels turned from near transparency to translucent maroon with the increase of immersing Fe^3+^ concentration or time. As revealed from Table 1, the water contents of T–Py–DN hydrogels slightly decreased with the increase of the pretreated FeCl_3(aq)_ concentrations, indicating the complex formation between ferric ions and carboxylate groups. The apparent colors of samples changed from translucent maroon to translucent with light yellow after being washed with EDTA solution (i.e., Figure S4b); elucidating ferric ions were extracted from the T–Fe–DN hydrogels.

### 3.3. Characteristic of Functional Groups within Hydrogels

**Figure 3:**
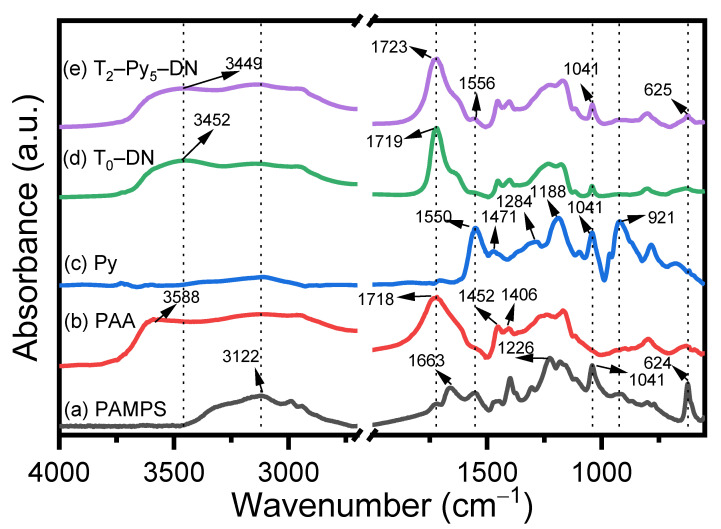
FT–IR spectra of (**a**) PAMPS; (**b**) PAA; (**c**) Py, (**d**) T_0_–DN, and (**e**) T_2_–Py_5_–DN samples.

FT–IR spectroscopy was utilized to analyze the difference in the functionality of hydrogels in each step. As shown in Figure 3a, the FT–IR spectrum of the PAMPS hydrogel represents the characteristic peaks at 3122, 1663, 1556, 1226, 1041, and 624 cm^−1^, corresponding to N–H stretching, secondary amide carbonyl groups stretching, secondary amide NH deformation, SO_2_ asymmetric stretch, SO_3_ symmetric stretch, and C–S stretch, respectively [48,49]. For FT–IR spectrum of PAA hydrogels, a broad band around 3451 cm^−1^ and a strong band at 1717 cm^−1^ are attributed by O–H stretching and C=O stretching of AA repeat units [50]. Besides, The CH_2_ deformation bands and C–O stretching coupled with OH in-plane bending are at 1452 and 1406 cm^−1^, respectively. In the case of Py, the characteristic bands at 1550 and 1470 cm^−1^ were acquired due to the C=C ring stretching of the pyrrole ring and C–N stretching vibration of the pyrrole ring, respectively [51]. Absorbance bands at 1284, 1188, and 1041 cm^−1^ are ascribed to C–H plane vibration, and the band at 921 cm^−1^ is for C–H out–of–plane vibration [52]. The FT–IR spectra of T_0_–DN and T_2_–Py_5_–DN hydrogels reveal similar characteristic absorbance bands, including the characteristic absorbance bands of PAA and PAMPS. A weak peak at 1556 cm^−1^ of the T_2_–Py_5_–DN hydrogel reveals the existence of the pyrrole C=C ring stretching in the sample. The resulting T–Py–DN hydrogels turned from brownish yellow to dark black after chemical oxidative reactions, indicating successful in situ polymerization of pyrrole monomers within T–Fe–DN scaffold.

### 3.4. Microstructures of DN Hydrogels

**Figure 4:**
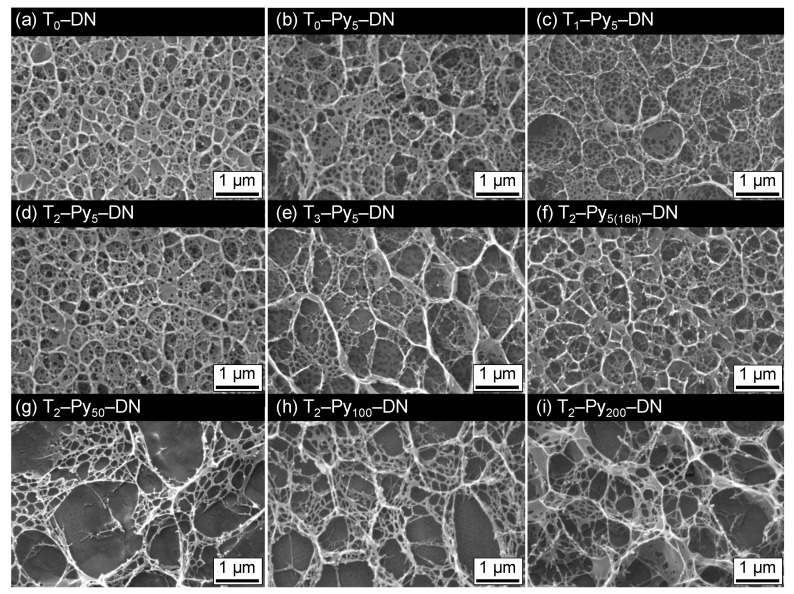
Cryo–SEM images of (**a**) T_0_–DN, (**b**) T_0_–Py_5_–DN, (**c**) T_1_–Py_5_–DN, (**d**) T_2_–Py_5_–DN, (**e**) T_3_–Py_5_–DN, (**f**) T_2_–Py_5(16h)_–DN, (**g**) T_2_–Py_50_–DN, (**h**) T_2_–Py_100_–DN, and (**i**) T_2_–Py_200_–DN hydrogels.

Ferric ions can be effectively coordinated with carboxylate groups of TOCN and PAA and result in physical crosslinking points within T–DNs. In this circumstance, the microstructures of T–DN and T–Py–DN hydrogels might be altered due to the absorption of Fe^3+^ and incorporations of Py chains. The microstructures of T–DN and T–Py–DN hydrogels were analyzed by cryo scanning electron microscopy (cryo–SEM) and presented in Figure 4. The cross-section of T_0_–DN hydrogel (i.e., Figure 4a) displayed homogeneous structures with numerous circular micropores with an average pore size of hundred nanometers. The main pores were likely the closed cells and represented similar size with an unambiguous wall but interconnected via the small pores. In T_0_–Py_5_–DN (i.e., Figure 4b), the interconnected microstructure was similar but the average pore size and distributions were obviously different. Larger (>1 μm) and more random pores in T_0_–Py_5_–DN hydrogel comparing to those in T_0_–DN hydrogel were observed, due to affect by the complexation among carboxylate groups and ferric ions. In T_1_–Py_5_–DN (i.e., Figure 4c), the microstructure was similar to the T_0_–Py_5_–DN, meaning 1wt.% TOCN in the DN did not significantly influence the microstructure. In T_2_–Py_5_–DN (i.e., Figure 4d), the microstructure displayed uniform micropores. In T_3_–Py_5_–DN (i.e., Figure 4e), the largest pores and obvious thicker walls were acquired among four samples of T_a_–Py_5_–DNs (a = 0, 1, 2, and 3). Presumably, 3 wt.% of TOCN performed or induced significant aggregations within the sample. Comparing the T_2_–Py_5_–DN and T_2_–Py_5(16h)_–DN (i.e., Figure 4d,f), longer pre-treating times of FeCl_3_ solutions did not significantly affect the microstructure. In T_1_–Py_50_–DN, T_1_–Py_100_–DN, and T_1_–Py_200_–DN (i.e., Figure 4g–i), however, high concentrations of incorporated ferric ions resulted in mesh-like structures. The irregular macroscopic structures might be ascribed to the increase of substantial complexation forces among carboxylate groups and ferric ions.

### 3.5. Thermal Stability of T–Py–DN Hydrogels

**Figure 5:**
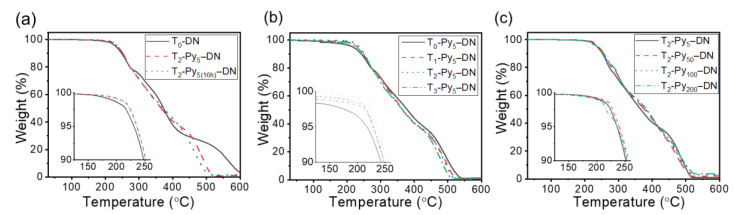
TGA traces of (**a**) T–DN and T–Py–DN hydrogels with various immersing time in 5 mM FeCl_3(aq)_, (**b**) T–Py–DN hydrogels with various TOCN contents, and (**c**) T–Py–DN treated with various FeCl_3(aq)_ concentrations.

Figure 5 shows thermogravimetric analysis (TGA) of three sets of dry samples in a nitrogen environment. The temperature at 5 wt.% loss of the samples was denoted *T*_d,5%_. The thermal stability profiles of all samples represented similar decomposition behaviors (*T*_d,5%_ = ca. 230–240 °C; char yield <4% at 500 °C). Nevertheless, T_0_–DN sample (i.e., the solid line in Figure 5a) represented the final decomposition stage (i.e., char yield < 4%) up to ca. 600 °C. Overall, with various TOCN contents (i.e., Figure 5b) or Fe^3+^ contents (i.e., Figure 5c), insignificant influences on thermal stability properties of T–Py–DN hydrogels were acquired. The corresponding thermal stability properties were summarized in Table 3.

### 3.6. Mechanical Properties of T–Py–DN Hydrogels

**Figure 6:**
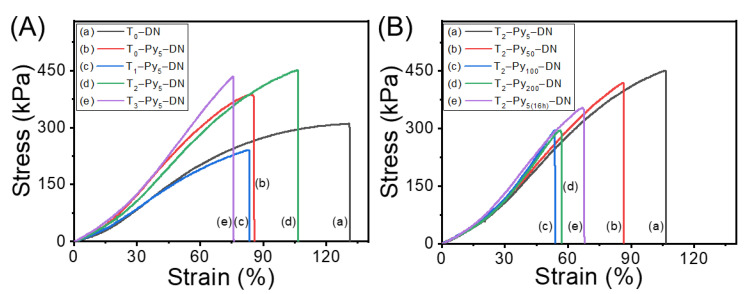
Stress-strain curves of various T–Py–DNs (**A**) with different TOCN contents and (**B**) pretreated with various immersing time or concentrations of FeCl_3(aq)_.

Universal tensile testing was further conducted to examine the mechanical strength of T–Py–DN hydrogels. Figure 6 demonstrates the tensile stress-strain curves of fully swollen hydrogels with various TOCN or Py contents. T_0_–DN (i.e., curve a in Figure 6A) represented a ductile property on the tensile strength and strain at break (i.e., σ_Tb_ = 310 kPa and ε_Tb_ = 130%). Comparing most of T_a_–Py_5_–DNs (i.e., curves b, d, and e in Figure 6A) to T_0_–DN, a brittle property with higher strength and lower strain at break (i.e., σ_Tb_ = ca. 390–450 kPa and ε_Tb_ = ca. 80–100%) was observed. No specific trends of mechanical properties were obvious with increases of TOCN content. While increasing the ferric ion contents (i.e., Figure 6B), interestingly, a gradual decrease trend of strains was observed from T_2_–Py_5_–DN, T_2_–Py_50_–DN, T_2_–Py_5(16h)_–DN, T_2_–Py_100_–DN, to T_2_–Py_200_–DN samples, illustrating a higher ferric ion concentration in the T–Py–DN hydrogels was unfavorable for the mechanical properties. The higher concentrations of Fe^3+^ within the hydrogels should lead to the less homogeneous microstructures and result in decrease of the mechanical properties. The microstructures can be revealed from the previous cryo-SEM as well. Overall, the breaking strength of T–Py–DN could be improved with incorporating the high strength TOCNs, but elongation property would be slightly sacrificed. From the results, an effective immersing concentration of FeCl_3(aq)_ and time for doping ferric ions was 5 mM and 0.5 h to carry out in situ chemical oxidative polymerization within the hydrogels. The mechanical properties were summarized in Table S1 (in supplementary materials).

### 3.7. Electrical Conductivity (Σ) and Resistivity (ρ) of T–Py–DN Hydrogels

**Figure 7:**
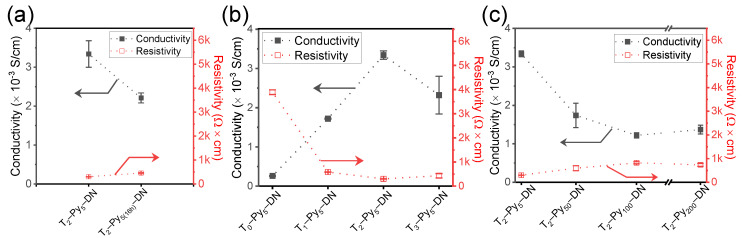
Measurements of T–Py–DNs of electrical conductivity and resistivity: (**a**) with various 5 mM FeCl_3(aq)_ pretreated times, (**b**) with various TOCN contents, and (**c**) with various pretreated FeCl_3(aq)_ concentrations.

Figure 7 presents the electrical conductivity (Σ) and resistivity of T–Py–DN hydrogels. We can expect the T_0_–DN has low conductivity due to a lack of conductive components (Σ(T_0_–DN) = 0.17 × 10^−3^ S/cm). With longer Fe^3+^ pre-treating times (i.e., Figure 7a), the conductivity decreased (Σ(T_2_–Py_5_–DN) = 3.34 × 10^−3^ S/cm; Σ(T_2_–Py_5(16h)_–DN) = 2.21 × 10^−3^ S/cm). In the absence of TOCN (i.e., Figure 7b), note that the conductivity was slightly enhanced by the incorporation of Py chains (Σ(T_0_–Py_5_–DN) = 0.26 × 10^−3^ S/cm). In the presence of TOCN, the conductivity further enhanced around one order. The best conductivity of T_2_–Py_5_–DN was acquired (Σ(T_2_–Py_5_–DN) = 3.34 × 10^−3^ S/cm) with 2 wt.% contents of TOCNs. Presumably the generated negative charges on the TOCN surfaces could effectively complex with Fe^3+^ ions in the presence of TOCN. Thus the cellulose nanostructures with the immobilized ferric complex can synergistically attain Py-rich micro-regions [33]. With increases of immersing Fe^3+^ concentrations of 5–200 mM (i.e., Figure 7c), the conductivity dropped down. This trend was similar to the results of mechanical properties that can be also revealed from the cyro-SEM analysis. Apparently, a finer mesh network structure has a higher conductivity. Thus, the optimal feeding amount of 2 wt.% TOCN was acquired, where the conductivity approached a maximum. It is possibly due to the microstructural uniform network within the T_2_–Py_5_–DN hydrogel (i.e., see the image of cryo–SEM in Figure 4c). The growth of conductive Py chains on the TOCN scaffolds was facilitated through electrostatic interactions and hydrogen bonding between Py and TOCN, leading to higher electrical conductivity. Table 3 summarizes the conductivity and resistivity results.

**Figure 8:**
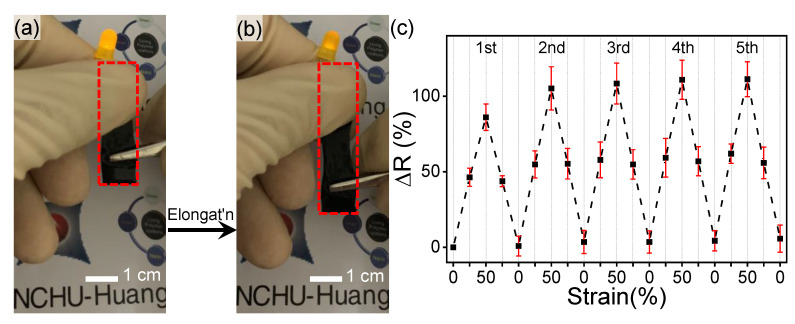
(**a**,**b**) Alternations of apparent light intensity on elongation. (**c**) Examinations of T_2_–Py_5_–DN hydrogel on Δ*R*(%) vs. strain (%) over elongation cycles.

A simple circuit module with a constant 3 V was connected to a light-emitting diode (LED) to examine the relationship between stretching and conducting of the T_2_–Py_5_–DN sample. As shown in Figure 8a, the LED was successfully lighted on and obtained good brightness. While stretched of T_2_–Py_5_–DN (i.e., Figure 8b), a dimmer light was observed. Then we tested the electrical resistance changes of the hydrogel to investigate the influence of elongation cycles on the electrical property at different strains (i.e., 0, 25, and 50%) of the T_2_–Py_5_–DN sample. The changes in electrical resistance (ΔR(%)) over elongation cycles were further estimated by the following equation:

$$∆R\;\left(\%\right)=\frac{R-R_{0}}{R_{0}}\times 100\%$$

where *R*_0_ is the original resistance of the fresh prepared hydrogel and R denotes the corresponding resistance at 0, 25, and 50% strains. The tensile stresses at 25 and 50% strains were around 85 and 225 kPa applied by a universal tensile testing machine. The relationship between electrical resistance (*R*) and resistivity (*ρ*) and can be calculated by:

$$R=\rho \frac{l}{A}$$

where *l* is the length of the hydrogel specimen and *A* is the cross-sectional area of the hydrogel specimen, assuming the intrinsic electrical resistivity and total volume of T_2_–Py_5_–DN hydrogel were constant during stretching. Figure 8c further represents our cycling tests based on resistance changes (Δ*R*(%)) versus different elongated strains. In the 1st stretching step with the strains of 25 and 50%, we acquired Δ*R*(%) = ca. 46 and 86%, correspondingly. In the next four “stretching” steps, the Δ*R*(%)s at 0, 25, and 50% strains slightly increased. The measured and estimated data of resistance changes further compared, T_2_–Py_5_–DN hydrogel was likely an isotropic material (see the ESI). The slight increment in the stretching steps is presumably due to the isotropic microstructure of the conductive parts being slightly damaged or disintegrated. In all “releasing” steps, however, the ΔR values can be all reversed back (i.e., toward total recovery of conductivity). These results indicated the designed T–Py–DN hydrogels not only possess tough and stretchable property but also perform reversible and adjustable conductivity.

## 4. Conclusions

Confirming by FT–IR analysis, PAMPS/PAA DN hydrogels hybridized with TOCN and Py were successfully prepared via a facile four-step procedure. *T*_d,5%_ of dry T–Py–DN samples were around 230–240 °C, indicating TOCN or Py did not significantly vary the thermal stability. The mechanical property was affected bydispersed TOCN or ferric ions. The Young’s modulus of all T–Py–DNs was similar (~1 MPa). T_2_–Py_5_–DN represented the best tensile strength and strain (i.e., σ_Tb_ = 451 kPa and ε_Tb_ = 107%). On the other hand, increases of the Fe^3+^ contents would result in less homogeneous microstructures (confirmed by cryo-SEM) and decrease the mechanical property. In the presence of TOCN, interestingly, good conductivity of T_2_–Py_5_–DN (Σ(T_2_–Py_5_–DN) = 3.34 × 10^−3^ S/cm) was acquired. This significant enhancement might be ascribed that the cellulose nanostructures with the immobilized ferric complex can synergistically attain Py-rich micro-regions. Eventually, structural uniform T_2_–Py_5_–DN performed reversible and adjustable electrical properties over several “stretching-and releasing” cycles. Last but not least, TOCN plays as a critical promoter toward obtainments of good performance T–Py–DN conductive hydrogels. We thus anticipate these conductive strong DN hydrogels have a high potential for a variety of applications in the field of intelligent electronics, neuro-transmission, or biomaterials.

## Figures and Tables

**Table 1:** Summary of contents of TOCN in Step 1, pretreatment FeCl_3(aq)_ concentrations in Step 3, water content, and degree of swelling for various T–Py–DN samples.

Sample	TOCN Content (wt.%)	[FeCl_3_]_0_ (mM)	Water Content (wt.%)	Degree of Swelling
T_0_–DN	0	–	94.2	16.4
T_0_–Py_5_–DN	0	5	93.5	14.6
T_1_–Py_5_–DN	1	5	92.7	12.8
T_2_–Py_5_–DN	2	5	94.4	16.2
T_3_–Py_5_–DN	3	5	93.2	13.8
T_2_–Py_5(16h)_–DN *	2	5	94.9	18.7
T_2_–Py_50_–DN	2	50	93.6	14.8
T_2_–Py_100_–DN	2	100	92.0	11.6
T_2_–Py_200_–DN	2	200	91.7	11.3

* The immersing time in 5 mM of FeCl_3(aq)_ for T_2_–DN pre-sample was 16 h.

**Table 2:** Concentrations of ferric ions extracted from T_2_–(Fe–)DN hydrogels.

Sample	Conc. of Fe^3+^ in DN Hydrogels (mM)
T_2_–DN	0
T_2_–Fe_5_–DN	3.68
T_2_–Fe_5(16h)_–DN	8.20
T_2_–Fe_50_–DN	6.97
T_2_–Fe_100_–DN	9.38
T_2_–Fe_200_–DN	20.67

**Table 3:** Summary of thermal stability (note: dry samples) and electrical conductivity/resistivity (note: fully swollen samples) of various T–Py–DN hydrogels.

Sample	*T*_d,5%_ (°C)	Char Yield (wt.%)	Conductivity (×10^−3^ S/cm)	Resistivity * (Ω × cm)
T_0_–DN	232	2.1	0.17	5930
T_0_–Py_5_–DN	228	1.6	0.26	3893
T_1_–Py_5_–DN	232	2.1	1.72	583
T_2_–Py_5_–DN	237	1.8	3.34	299
T_3_–Py_5_–DN	233	1.3	2.37	431
T_2_–Py_5(16h)_–DN	237	1.8	2.21	454
T_2_–Py_50_–DN	241	2.5	1.74	594
T_2_–Py_100_–DN	237	3.6	1.22	823
T_2_–Py_200_–DN	238	3.9	1.37	736

* The value of resistivity was calculated from the reciprocal of the conductivity.

## Supplementary Materials

The following are available online at https://www.mdpi.com/2073-4360/12/12/2835/s1, Estimation of the resistance change of hydrogels; Table S1: Summary of tensile testing results of T–Py–DN samples; Figure S1: (a) UV–Vis spectra of Fe(III)EDTA solutions with different concentrations and (b) linear fitting of the UV–Vis absorbance at 260 nm versus the concentration of Fe(III)EDTA aqueous; Figure S2: SEM images of (a) original cellulose and (b) TOCN. The counts of fiber numbers versus the diameter of (c) cellulose fibers and (d) nanofibrils from the SEM images of (a,b), respectively; Figure S3: UV–Vis spectra of the Fe(III)EDTA solutions extracted from T_2_–(Fe–)DN hydrogels; Figure S4: Apparent color changes of various T–Fe–DN hydrogels after (a) immersing in various FeCl_3(aq)_ with different time and (b) removing ferric ions with EDTA solutions.
