# Supplementary material for: Preparations of Tough and Conductive PAMPS/PAA Double Network Hydrogels Containing Cellulose Nanofibers and Polypyrroles
Source: Polymers (Basel). 2020 Nov 28;12(12):2835. doi: 10.3390/polym12122835 (PMC7760924; doi:10.3390/polym12122835)
Supplement: Supplementary file 1 [file polymers-12-02835-s001.pdf]

polymers

Electronic Supplement Information (ESI) for

Preparations of Tough and Conductive PAMPS/PAA Double Network Hydrogels Containing Cellulose Nanofibers and Polypyrroles

Cheng-Wei Tu[1], Fang-Chang Tsai[2,*] Jem-Kun Chen[3], Huei-Ping Wang[4], Rong-Ho Lee[4], Jiawei Zhang[5], Tao Chen[5], Chung-Chi Wang[6] and Chih-Feng Huang[4,*]

[1] Industrial Technology Research Institute, Chutung, Hsinchu 31057, Taiwan; CWTu@itri.org.tw (C.-W.T.)
[2] Hubei Key Laboratory of Polymer Materials, Key Laboratory for the Green Preparation and Application of Functional Materials (Ministry of Education), Hubei Collaborative Innovation Center for Advanced Organic Chemical Materials, School of Materials Science and Engineering, Hubei University, Wuhan 430062, China
[3] Department of Materials Science and Engineering, National Taiwan University of Science and Technology, Taipei 10607, Taiwan; jkchen@mail.ntust.edu.tw (J.-K.C.)
[4] Department of Chemical Engineering, i-Center for Advanced Science and Technology (iCAST), National Chung Hsing University, Taichung 40227, Taiwan; ac9562@gmail.com (H.-P.W.); rhl@nchu.edu.tw (R.-H.L.)
[5] Key Laboratory of Marine Materials and Related Technologies, Zhejiang Key Laboratory of Marine Materials and Protective Technologies, Ningbo Institute of Materials Technology and Engineering, Chinese Academy of Sciences, Ningbo 315201, China; zhangjiawei@nimte.ac.cn (J.Z.); tao.chen@nimte.ac.cn (T.C.)
[6] Division of Cardiovascular Surgery, Veterans General Hospital, Taichung 40705, Taiwan; chungchi@vghtc.gov.tw (C.-C.W.)

* Correspondence: tfc0323@gmail.com (F.-C.T.); HuangCF@dragon.nchu.edu.tw (C.-F.H.)

Captions:

Table S1. Summary of tensile testing results of T–Py–DN samples.

Fig. S1. (a) UV–Vis spectra of Fe(III)EDTA solutions with different concentrations and (b) linear fitting of the UV–Vis absorbance at 260 nm versus the concentration of Fe(III)EDTA aqueous.

Fig. S2. SEM images of (a) original cellulose and (b) TOCN. The counts of fiber numbers versus the width of (c) cellulose fibers and (d) nanofibrils from the SEM images of (a) and (b), respectively.

Fig. S3. UV–Vis spectra of the Fe(III)EDTA solutions extracted from T_2–(Fe–)DN hydrogels.

Fig. S4. Apparent color changes of various T–Fe–DN hydrogels after (a) immersing in various $FeCl_{3(aq)}$ with different time and (b) removing ferric ions with EDTA solutions.

1. Estimation of the resistance change of hydrogels.

Assuming constant resistivity and volume for T_2–Py_5–DN hydrogel, a relationship between ΔR (%) and the strain (ε in %) can be derived for the case of ideal homogeneous deformation of the sample. Because the total volume is constant during stretching, the value of cross-section area (A) multiples length (l) of the hydrogel specimen is constant (i.e. $l \times A = l_0 \times A_0$). Where l_0 and A_0 are the original length and cross-section area of the hydrogel specimen. The resistance change could be derived to the fallowing equation:

$$\Delta R\ (\%) = \left(\frac{l^2}{{l_0}^2} - 1\right) \times 100\%$$

Replace l/l_0 by $(1+\varepsilon/100)$ and the resistance change can further derive to:

$$\Delta R\ (\%) = \frac{\varepsilon^2}{100} + 2\varepsilon$$

Thus, the resistance change was estimated and expected to be observed at strain ratio of 0–25–50 %, respectively. For the first elongation cycle at strain ratio of 25 and 50 %, slightly deviations around 46 and 86% of resistance change were measured. However, the resistance changes at 0–25–50 % strain ratio slightly increase at 2–5 cycles. The final electrical resistance changes during releasing at 5th cycle are around 111, 56, and 6 % relevant to the strain ratio of 50–25–0 %, respectively, which were close to the abovementioned estimated results of resistance change. The data of electrical resistance changes over elongation cycles indicated the hydrogel was isotopic.

Table S1. Summary of tensile testing results of T–Py–DN samples.

Sample	Stress (kPa)	Strain (%)	Modulus (MPa)	Toughness* (kJ/m^3)
T_0–DN	310.6	131.3	0.93	248.4
T_2–Py_5–DN	451.1	106.7	0.96	249.1
T_2–$Py_{5(16h)}$–DN	354.4	67.8	0.96	112.9
T_0–Py_5–DN	386.1	85.8	1.15	170.2
T_1–Py_5–DN	239.6	83.6	0.68	104.8
T_3–Py_5–DN	434.6	76.0	1.34	145.8
T_2–Py_{50}–DN	418.9	86.6	0.96	172.8
T_2–Py_{100}–DN	295.2	54.1	0.98	63.8
T_2–Py_{200}–DN	295.6	54.0	0.94	69.9

* Toughness was estimated from the area of the obtaining stress-strain curve.

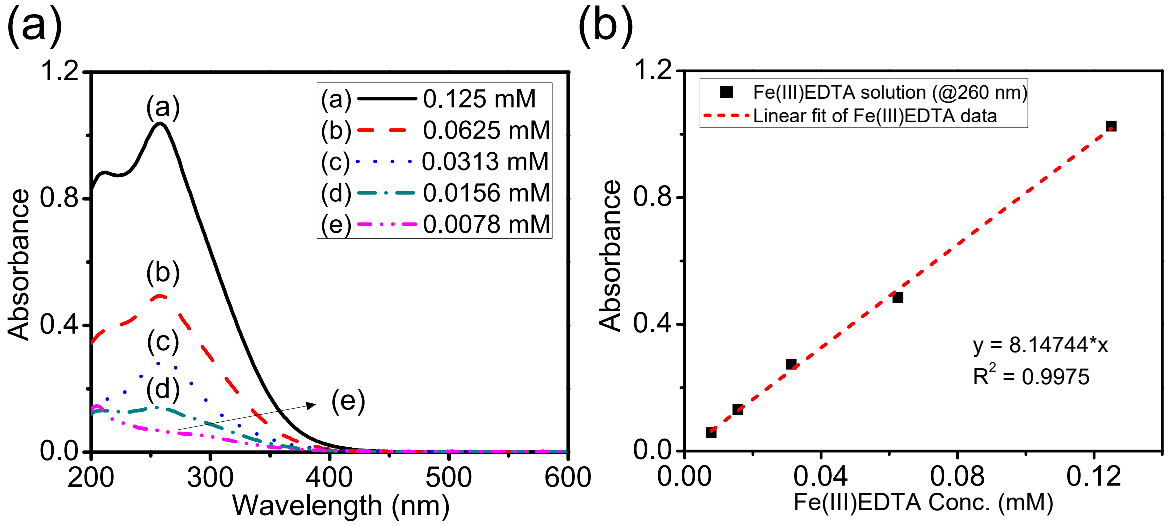

Figure S1. (a) UV–Vis spectra of Fe(III)EDTA solutions with different concentrations and (b) linear fitting of the UV–Vis absorbance at 260 nm versus the concentration of Fe(III)EDTA aqueous.

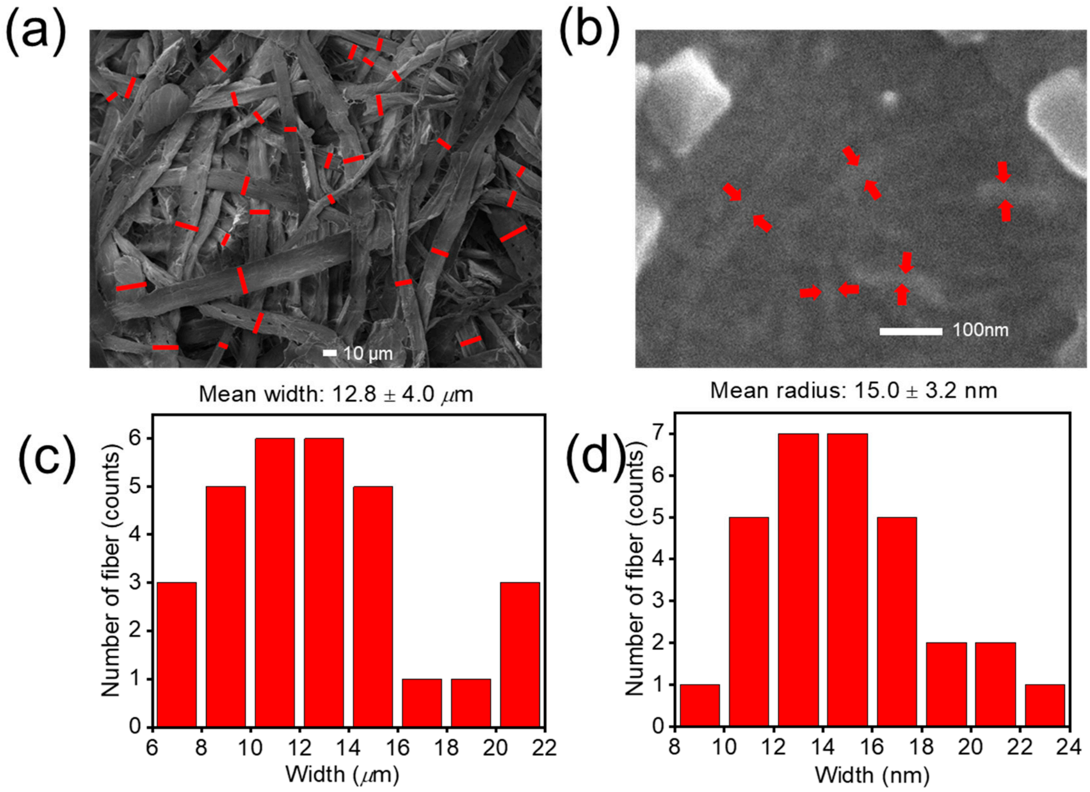

Figure S2. SEM images of (a) original cellulose and (b) TOCN. The counts of fiber numbers versus the width of (c) cellulose fibers and (d) nanofibrils from the SEM images of (a) and (b), respectively.

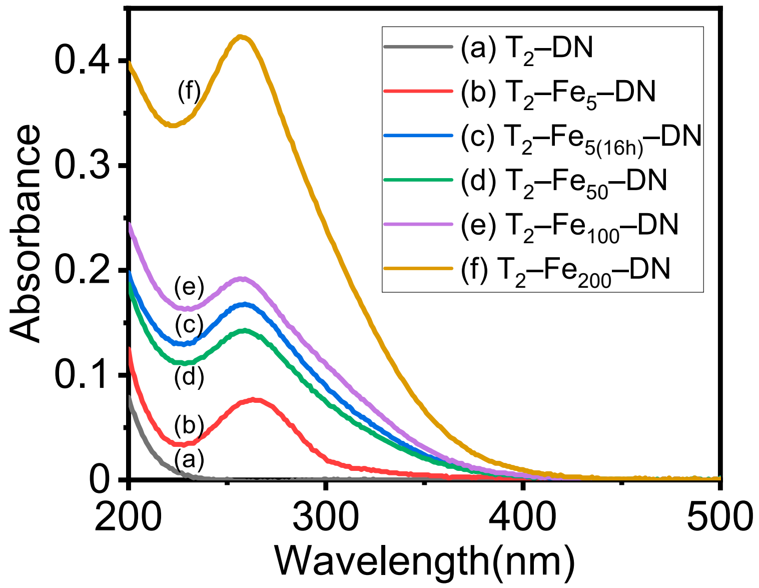

Figure S3. UV–Vis spectra of the Fe(III)EDTA solutions extracted from T_2–(Fe–)DN hydrogels.

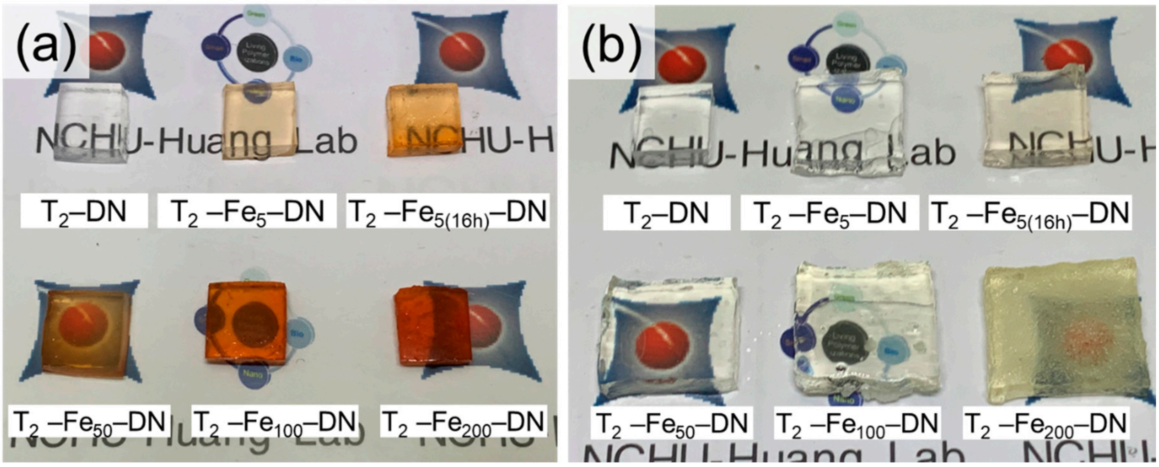

Figure S4. Apparent color changes of various T–Fe–DN hydrogels after (a) immersing in various $FeCl_{3(aq)}$ with different time and (b) removing ferric ions with EDTA solutions.

© 2020 by the authors. Licensee MDPI, Basel, Switzerland. This article is an open access article distributed under the terms and conditions of the Creative Commons Attribution (CC BY) license (http://creativecommons.org/licenses/by/4.0/).
